# Cross-sectional and prospective associations between children's eating behavior and visceral fat area and trunk fat mass

**DOI:** 10.3389/fped.2024.1514535

**Published:** 2024-12-12

**Authors:** Youxin Wang, Fangjing Shen, Pingping Zhang, Miao Xu, Danqi Qiu, Hui Wang, Li Li

**Affiliations:** ^1^ Department of Endocrinology and Metabolism, The First Affiliated Hospital of Ningbo University, Ningbo, Zhejiang, China; ^2^ Department of Maternal and Child Health, School of Public Health, Peking University, Beijing, China; ^3^ Ningbo Center for Healthy Lifestyle Research, The First Affiliated Hospital of Ningbo University, Ningbo, Zhejiang, China

**Keywords:** children, abdominal fat, obesity, overweight, eating behaviors

## Abstract

**Background:**

Understanding children's eating behaviors is crucial for elucidating the etiology of childhood obesity. However, the relationship between these behaviors and abdominal fat accumulation remains poorly understood. This study aims to investigate this association in primary school children.

**Methods:**

This study included 1,475 children aged 8–10 years in Ningbo, China. Body composition was measured both at baseline (September 2022) and after 9 months of follow-up (June 2023). Primary caregivers completed the Children's Eating Behavior Questionnaire (CEBQ). A mixed-effects linear model was applied to analyze the relationships between children's eating behaviors and body composition.

**Results:**

Greater visceral fat area (VFA) was associated with greater slowness in eating [β = 0.25 (0.02, 0.49)], greater food responsiveness [β = 0.29 (0.14, 0.43)], increased emotional undereating [β = 0.35 (0.17, 0.54)], and more emotional overeating [β = 0.21 (0.02, 0.39)]. Children with greater trunk fat mass (TrFM) tended to have greater food responsiveness [β = 0.02 (0.01, 0.03)] and greater enjoyment of food [β = 0.01 (0.00, 0.03)], increased emotional undereating [β = 0.02 (0.01, 0.03)] and emotional overeating [β = 0.02 (0.00, 0.03)]. Prospectively, positive correlations were observed between VFA and food responsiveness [β = 0.18 (0.02, 0.35)], as were emotional undereating [β = 0.31 (0.10, 0.52)] and emotional overeating [β = 0.24 (0.03, 0.46)]. Similarly, TrFM was positively correlated with food responsiveness [β = 0.02 (0.00, 0.03)], enjoyment of food [β = 0.02 (0.00, 0.04)], emotional undereating [β = 0.02 (0.01, 0.04)] and emotional overeating [β = 0.02 (0.01, 0.04)].

**Conclusions:**

Our findings suggest that eating behaviors are significantly associated with abdominal fat accumulation in primary school children. Addressing specific eating behaviors may be crucial in mitigating abdominal fat and its related health risks.

## 1 Introduction

In recent years, the increase in childhood obesity has become a critical global health concern (1). In China, studies have shown that the average waist circumference of children and adolescents increased from 60.27 cm in 1993–64.31 cm in 2015 (2). Between 2015 and 2019, 11% of children and adolescents aged 6–17years were overweight and 7.9% were obese (3). This trend has serious health implications, as obesity is closely linked to cardiovascular diseases, type 2 diabetes, and hypertension (4, 5).

Body mass index (BMI), although widely used to measure obesity, often fails to distinguish between muscle and adipose tissue. A certain number of children may have a higher BMI because of increased muscle rather than excessive fat accumulation (6). Given the complexity of obesity phenotypes, various modifiable risk factors influence not only BMI but also abdominal fat distribution (7). Additionally, visceral fat (8) and trunk fat mass (TrFM) (9), measured through bioelectrical impedance analysis, provide more accurate indicators of abdominal fat accumulation than waist circumference alone. Excessive visceral and trunk fat are closely associated with multiple health risks, and studies suggest that increased visceral fat area (VFA) predicts cardiovascular complications and metabolic health issues in children and adolescents (10, 11). TrFM has also been linked to increased cardiovascular metabolic risk (12), making a reduction in visceral and trunk fat key objectives in obesity intervention.

Children's eating behaviors influence obesity (13, 14). The Children's Eating Behavior Questionnaire (CEBQ) is a comprehensive tool comprising 35 items organized into 8 subscales, which has been widely used to evaluate eating behaviors in children aged 2–13 years and has demonstrated good internal consistency, test-retest reliability, and reasonable construct validity in China (15). The subscales cover both food “approach” behaviors, such as food response, enjoyment of food, emotional overeating, and desire to drink as well as food “avoidance’ behaviors, including satiety response, slowness in eating, fussiness and emotional undereating (16). Higher scores in food “approach” behaviors among children who are overweight, obesity or have a high body fat percentage corresponded to lower scores in food “avoidance” behavior (13, 17). In a cross-sectional study, children exhibiting greater food responsiveness and enjoyment tended to have higher BMIs, whereas emotional undereating, satiety and fussiness were negatively correlated with BMI (18). Additionally, a linear regression analysis revealed a positive association between childhood obesity and food responsiveness and the desire to drink, with a negative correlation observed for satiety. Power et al. proposed a longitudinal bidirectional relationship between emotional overeating and weight status in 8-year-old Hispanic children (19). However, research on obese children in Thailand has not revealed a clear link between children's eating behavior and BMI (20).

Notably, most studies are cross-sectional and use BMI to assess childhood obesity, with unclear relationships between children's eating behavior and abdominal fat accumulation. Cross-sectional studies are limited by reverse causality, and more prospective or longitudinal studies are needed to establish causal pathways. This study aims to fill these gaps by examining both cross-sectional and prospective associations between children's eating behaviors, visceral fat, and trunk fat mass.

## 2 Materials and methods

### 2.1 Participants

**Figure 1:**
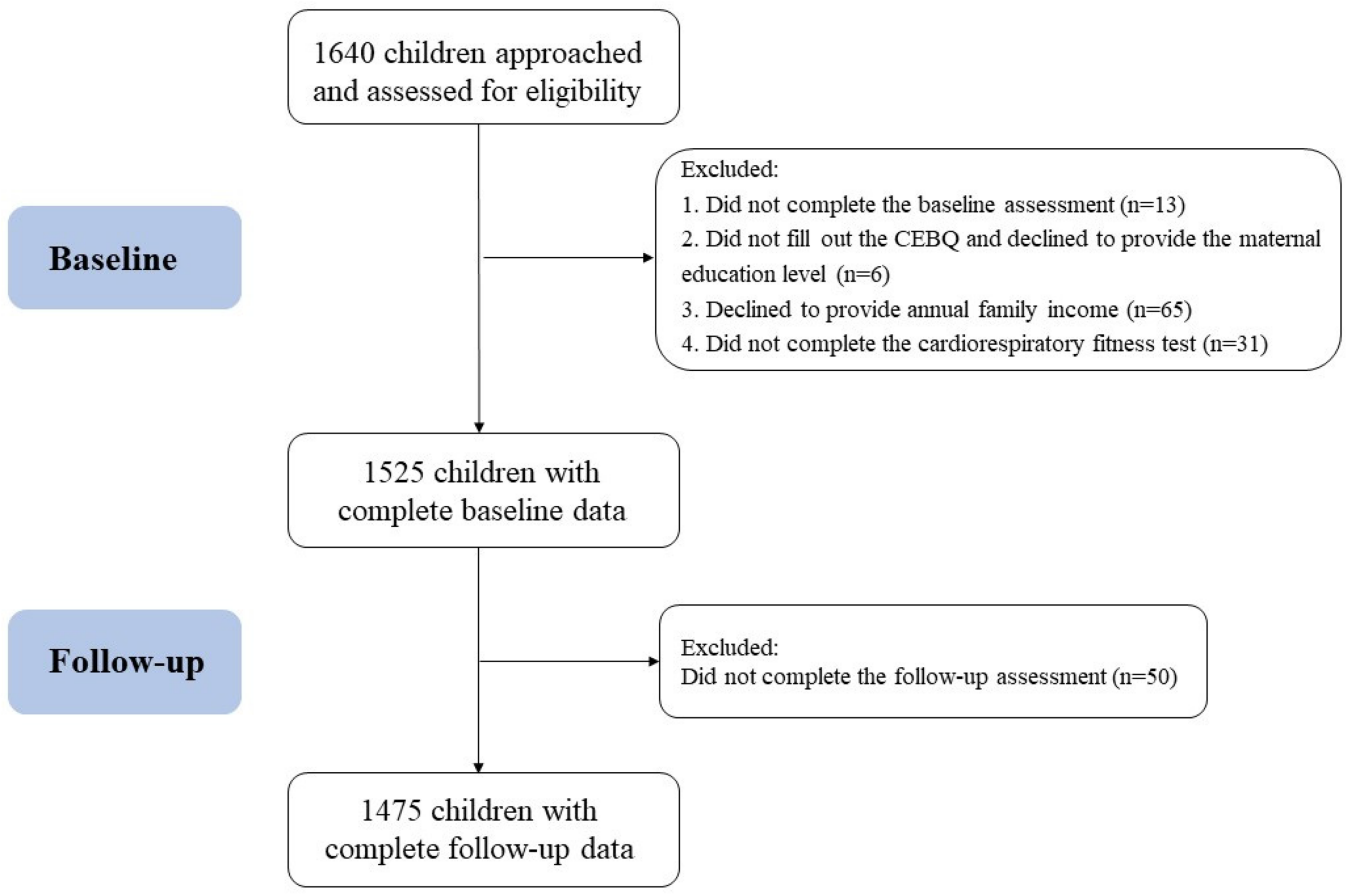
Flowchart of the participants examined in the present study.

The present study conducted an analysis utilizing data from the “Optimizing Intervention Effects in Children and Adolescents” (OptiChild) program (Registration No: NCT05482165). A total of 1,640 children from Ningbo City, Zhejiang Province, China, were recruited for the study. The flowchart illustrating the selection of the study population in the current study is depicted in Figure 1. The program received approval from the Ethics Committee of the First Affiliated Hospital of Ningbo University (Approval No. 2021-R168). Written informed consent was obtained from all the participating students and their primary guardians.

### 2.2 Measurements

#### 2.2.1 Demographics

The parents of the participants completed a questionnaire, providing data on their children's birth, sex, maternal education level (under high school or high school or above), and annual family income (≤100 thousand, 100–300 thousand, 300–500 thousand or >500 thousand). In addition, each participant completed a 20-meter shuttle run test under the guidance of a physical education teacher trained by the project team and recorded the total number of laps completed to evaluate cardiorespiratory fitness (CRF) (21, 22).

#### 2.2.2 CEBQ

The parents of the participants completed the children's eating behavior questionnaire at baseline (CEBQ). The CEBQ comprises 35 items and includes eight subscales: Satiety responsiveness (SR) (e.g., “My child has a big appetite”), slowness in eating (SE) (e.g., “My child eats slowly”), fussiness (FU) (e.g., “My child refuses new foods at first”), food responsiveness (FR) (e.g., “My child's always asking for food”), enjoyment of food (EF) (e.g., “My child enjoys eating”), desire to drink (DD) (e.g., “If given the chance, my child would drink continuously throughout the day”), emotional undereating (EU) (e.g., “My child eats less when s/he is upset”), and emotional overeating (EO) (e.g., “My child eats more when anxious”). Parents reported their children's eating behavior on a 5-point Likert scale ranging from “never” to “always”, where higher scores indicate a greater frequency of eating behavior on each subscale (16). In this study, data from these eight subscales are reported.

#### 2.2.3 The measurements of BMI, VFA, and TrFM

Weight, VFA and TrFM were measured via a bioimpedance analyzer (Inbody 770, California, USA), which has been previously validated for accuracy and reliability. The participants followed the system's instructions by wearing only a single-layered shirt and shorts, standing barefoot on the designated analyzer plates. They maintained a natural posture with their arms hanging by their sides, holding the electrodes and placing their thumbs on them. The body composition test lasted between 2 and 3 min. Height was measured via a mechanical height meter (Stadiometer) with participants not wearing shoes. BMI was calculated as weight (kg) divided by height squared (m^2^).

### 2.3 Data analysis

Participants with complete data were included in the analysis. Descriptive analyses were conducted for the characteristics of the participants and are presented as the means. To determine the associations between the CEBQ scales and VFA and TrFM, a mixed effect model was utilized. This model included school-level random intercepts to account for the correlation due to the clustering of children within schools, as the survey was conducted in units of schools. In Model 2 of the cross-sectional analysis, we adjusted for age sex and BMI. In Model 3, we further adjusted for maternal education level, annual family income, and CRF. In the prospective analysis (23), we corrected for the random effects of school, age, sex, BMI, maternal education level, annual family income, CRF and intervention group. Furthermore, a sensitivity analysis was conducted using the 9-month follow-up data on CEBQ scores, VFA, and TrFM, to assess the robustness of the cross-sectional associations. A *P*-value <0.05 was considered to indicate statistical significance. All the statistical analyses were performed via R4.3.0 (R Core Team).

## 3 Results

### 3.1 Participant characteristics

A total of 1,475 children, with a mean age of 8.5 ± 0.3 years (779 boys and 696 girls), were included in the analysis. The mean BMI was 16.5 ± 2.6 kg/m2. The majority of the children's mothers had at least a high school education (68.9%), and nearly half of the families (43.1%) reported an annual income between 100,000 and 300,000 RMB. Table 1 presents the children's VFA and TrFM at baseline and after 9 months of follow-up, along with the CEBQ scores for the eight dimensions at baseline.

**Table 1:** Descriptives statistics of the final sample.

Characteristic	Study sample (*n* = 1,475)
Age (years)	8.5 (0.3)
Sex, *n* (%)	
Boy	779 (52.8)
Girl	696 (47.2)
Maternal education level, *n* (%)	
Under high school	458 (31.1)
High school or above	1,017 (68.9)
Annual family income, *n* (%)	
≤ 100 thousand	260 (17.6)
100–300 thousand	636 (43.1)
300–500 thousand	361 (24.5)
> 500 thousand	218 (14.8)
BMI (kg/cm^2^)	16.5 (2.6)
CRF, (ml/kg/min.)	48.4 (3.4)
CEBQ, score	
SR	14.6 (2.3)
SE	11.0 (2.0)
FU	17.6 (2.2)
FR	12.0 (3.4)
EF	12.6 (2.7)
DD	8.3 (1.9)
EU	11.0 (2.6)
EO	9.0 (2.5)
Body composition in abdomen at baseline	
TrFM, (kg)	2.0 (2.2)
VFA, (cm^2^)	28.4 (18.7)
Body composition in abdomen at 9 months’ follow up	
TrFM, (kg)	2.3 (2.3)
VFA, (cm^2^)	30.2 (19.9)

BMI, body mass index; CRF, cardiorespiratory fitness; CEBQ, children's eating behavior questionnaire; SR, satiety responsiveness; SE, slowness in eating; FU, fussiness; FR, food responsiveness; EF, enjoyment of food; DD, desire to drink; EU, emotional undereating; EO, emotional overeating; TrFM, trunk fat mass; VFA, visceral fat area.

### 3.2 Cross-sectional associations between the CEBQ score and VFA

Table 2 presents the cross-sectional associations between VFA, TrFM and each CEBQ score. After adjusting for the random effect of school (Model 1), significant positive correlations were observed between children's VFA and TrFM with FR (β = 1.59 [1.32, 1.86] and β = 0.19 [0.16, 0.22), EF [β = 2.33 [2.00, 2.66] and β = 0.30 [0.27, 0.34]], DD [β = 1.36 [0.86, 1.86] and β = 0.18 [0.12, 0.24]] and EO [β = 1.06 [0.68,1.43] and β = 0.13 [0.08, 0.17]]. Furthermore, VFA and TrFM were negatively correlated with SR [β = −0.55 [−0.96, −0.13] and β = −0.08 [−0.13, −0.03]] and SE [β = −1.52 [−1.99, −1.06] and β = −0.22 [−0.27, −0.17]]. After adjusting for confounding factors such as age, sex, and BMI (Model 2), the mixed effect model revealed persistent cross-sectional associations between VFA and SE [0.29 (0.05, 0.53)], FR [0.26 (0.11, 0.41)], and EO [0.20 (0.01, 0.38)]. The TrFM remained associated with FR [0.02 (0.01, 0.03)] and EO [0.02 (0.00, 0.03)]. Additionally, after further adjustment for age, sex, and BMI, VFA and TrFM were positively correlated with EU [β = 0.35 [0.16, 0.53] and β = 0.02 [0.01, 0.04]]. When further adjustments were made for maternal education level, annual family income and CRF (Model 3), the results remained consistent with those observed in Model 2.

**Table 2:** Relationships between VAF, trFM and CEBQ scores in children at baseline.

Body composition	CEBQ scales	Model1^a^ β (95%CI)	*P*-value	Model2^b^ β (95%CI)	*P*-value	Model3^c^ β (95%CI)	*P*-value
VFA	SR	−0.55 (−0.96, −0.13)	0.01*	0.13 (−0.08, 0.33)	0.23	0.12 (−0.08, 0.33)	0.24
	SE	−1.52 (−1.99, −1.06)	<0.001*	0.29 (0.05, 0.53)	0.02*	0.25 (0.02, 0.49)	0.04*
	FU	0.33 (−0.11, 0.77)	0.14	0.21 (−0.01, 0.43)	0.06	0.20 (−0.02, 0.42)	0.07
	FR	1.59 (1.32,1.86)	<0.001*	0.26 (0.11, 0.41)	<0.001*	0.29 (0.14, 0.43)	<0.001*
	EF	2.33 (2.00,2.66)	<0.001*	0.03 (−0.16, 0.22)	0.76	0.07 (−0.12, 0.25)	0.49
	DD	1.36 (0.86,1.86)	<0.001*	−0.07 (−0.33, 0.18)	0.58	−0.01 (−0.26, 0.24)	0.92
	EU	0.03 (−0.34, 0.40)	0.89	0.35 (0.16, 0.53)	<0.001*	0.35 (0.17, 0.54)	<0.001*
	EO	1.06 (0.68,1.43)	<0.001*	0.20 (0.01, 0.38)	0.04*	0.21 (0.02, 0.39)	0.03*
TrFM	SR	−0.08 (−0.13, −0.03)	<0.001*	0.01 (−0.01, 0.02)	0.35	0.01 (−0.01, 0.02)	0.41
	SE	−0.22 (−0.27, −0.17)	<0.001*	0.01 (−0.01, 0.03)	0.30	0.01 (−0.01, 0.02)	0.53
	FU	0.03 (−0.02, 0.08)	0.26	0.01 (−0.00, 0.03)	0.09	0.01 (−0.00, 0.03)	0.12
	FR	0.19 (0.16, 0.22)	<0.001*	0.02 (0.01, 0.03)	<0.001*	0.02 (0.01, 0.03)	<0.001*
	EF	0.30 (0.27, 0.34)	<0.001*	0.01 (−0.00, 0.02)	0.13	0.01 (0.00, 0.03)	0.05*
	DD	0.18 (0.12, 0.24)	<0.001*	−0.00 (−0.02, 0.02)	0.75	0.00 (−0.02, 0.02)	0.80
	EU	−0.02 (−0.06, 0.02)	0.41	0.02 (0.01, 0.04)	<0.001*	0.02 (0.01, 0.03)	<0.001*
	EO	0.13 (0.08, 0.17)	<0.001*	0.02 (0.00, 0.03)	0.03*	0.02 (0.00, 0.03)	0.02*

CEBQ, children's eating behavior questionnaire; VFA, visceral fat area; TrFM, trunk fat mass; SR, satiety responsiveness; SE, slowness in eating; FU, fussiness; FR, food responsiveness; EF, enjoyment of food; DD, desire to drink; EU, emotional undereating; EO, emotional overeating.

^a^ Model 1: Adjusted only for school as a random effect in the mixed effect model.

^b^ Model 2: Adjusted for school as a random effect; age, sex and BMI in the mixed effect model.

^c^ Model 3: Adjusted for school as a random effect; age, sex, BMI, maternal education level, annual family income and CRF.

* Indicates statistical significance.

### 3.3 Prospective associations between the CEBQ score and VFA and TrFM

As presented in Table 3, after adjusting for school as a random effect, as well as age, sex, BMI, maternal education level, annual family income, CRF and intervention group, prospectively positive correlations were observed between VFA and FR [0.18 (0.02, 0.35)], EU [0.31 (0.10, 0.52)] and EO [0.24 (0.03, 0.46)]. Similarly, TrFM was positively correlated with FR [0.02 (0.00, 0.03)], EF [0.02 (0.00, 0.04)], EU [0.02 (0.01, 0.04)] and EO [0.02 (0.01, 0.04)].

**Table 3:** Relationships between baseline CEBQ and VFA and trFM at 9 months of follow-up in children.

Body composition	CEBQ scales	β (95%CI)^a^	*P*-value
VFA	SR	0.10 (−0.13, 0.34)	0.38
	SE	0.11 (−0.16, 0.39)	0.41
	FU	0.11 (−0.14, 0.36)	0.39
	FR	0.18 (0.02, 0.35)	0.03*
	EF	0.10 (−0.11, 0.32)	0.35
	DD	0.13 (−0.16, 0.42)	0.38
	EU	0.31 (0.10, 0.52)	<0.001*
	EO	0.24 (0.03, 0.46)	0.03*
TrFM	SR	0.01 (−0.01, 0.03)	0.35
	SE	−0.00 (−0.03, 0.02)	0.73
	FU	0.01 (−0.01, 0.03)	0.45
	FR	0.02 (0.00, 0.03)	0.03*
	EF	0.02 (0.00, 0.04)	0.01*
	DD	0.01 (−0.01, 0.04)	0.28
	EU	0.02 (0.01, 0.04)	0.01*
	EO	0.02 (0.01, 0.04)	0.01*

CEBQ, children's eating behavior questionnaire; VFA, visceral fat area; TrFM, trunk fat mass; SR, satiety responsiveness; SE, slowness in eating; FU, fussiness; FR, food responsiveness; EF, enjoyment of food; DD, desire to drink; EU, emotional undereating; EO, emotional overeating.

^a^ Adjusted for school as random effect, age, sex, baseline BMI, maternal education level, annual family income, baseline CRF and intervention groups.

* Indicates statistical significance.

### 3.4 Sensitivity analysis

A sensitivity analysis was conducted on the complete data set from 1,485 children after 9 months of follow-up. Adjustments for group, baseline age, sex, maternal education level, annual family income, BMI at 9 months, and CRF were made. As presented in Table 4, the results remained consistent with those observed at baseline, with significant positive correlations found between SE [β = 0.37 (0.12, 0.62)], FR [β = 0.23 (0.09, 0.38)] and VFA. Additionally, significant positive correlations were observed between FR [β = 0.02 (0.01, 0.03)], EF [β = 0.02 (0.00, 0.03)] and TrFM. Other outcomes showed similar trends.

**Table 4:** Relationships between VFA, trFM and the CEBQ in children after 9 months of follow-up.

Body composition	CEBQ scales	β (95%CI)^a^	*P*-value
VFA	SR	−0.06 (−0.28, 0.16)	0.58
	SE	0.37 (0.12, 0.62)	0.004*
	FU	0.21 (−0.02, 0.43)	0.08
	FR	0.23 (0.09, 0.38)	0.002*
	EF	0.14 (−0.05, 0.33)	0.14
	DD	0.02 (−0.23, 0.26)	0.86
	EU	−0.03 (−0.22, 0.16)	0.73
	EO	0.04 (−0.15, 0.24)	0.68
TrFM	SR	−0.01 (−0.02, 0.01)	0.29
	SE	0.02 (0.00, 0.04)	0.02*
	FU	0.01 (−0.01, 0.03)	0.22
	FR	0.02 (0.01, 0.03)	<0.001*
	EF	0.02 (0.00, 0.03)	0.008*
	DD	0.00 (−0.02, 0.02)	0.85
	EU	0.00 (−0.01, 0.01)	0.99
	EO	0.01 (−0.01, 0.02)	0.37

CEBQ, children's eating behavior questionnaire; VFA, visceral fat area; TrFM, trunk fat mass; SR, satiety responsiveness; SE, slowness in eating; FU, fussiness; FR, food responsiveness; EF, enjoyment of food; DD, desire to drink; EU, emotional undereating; EO, emotional overeating.

^a^ Adjusted for school as random effect, intervention groups, baseline age, sex, maternal education level, annual family income, BMI and CRF at 9 months.

* Indicates statistical significance.

## 4 Discussion

To our knowledge, this study is the first to comprehensively examine both cross-sectional and prospective associations between children's eating behaviors and abdominal fat accumulation. Broadly, increases in VFA were associated with higher scores in FR, EU, and EO, whereas increases in TrFM were linked to elevated scores in FR, EF, EU and EO.

Cross-sectionally, we found that VFA and TrFM were positively correlated with FR, EF, DD and EO, but negatively correlated with SR and SE. These findings align with the literature, which has consistently reported that obese children exhibit greater food responsiveness and poorer dietary self-regulation than their healthy-weight peers do (13, 24). This heightened food responsiveness may be driven by increased sensitivity to external cues such as the sight and smell of food, particularly high-sugar and high-fat options, which can lead to overeating even in the absence of hunger (25). For example, a similar study conducted among adolescents in Ohio revealed positive correlations between food responsiveness and emotional overeating with higher cardiovascular metabolic risk scores, including increased VFA (26). Additionally, Tay et al. conducted a sex-stratified analysis and reported that only DD was associated with increased waist circumference among girls (27). This observation may be explained by previous research indicating that girls aged 9–14 years tend to have higher total sugar intake than boys do (28).

Consistent with expectations, SR and SE typically exhibit negative correlations with childhood obesity (29, 30). Children with a high satiety response tend to follow the brain's cues and stop eating when they are full. For example, if they have snacks before a meal, they may not finish the meal. The link between eating speed and obesity is also physiologically plausible. Eating quickly can lead to the consumption of more food overall, increasing total energy intake (31, 32). Previous studies have shown that fast eating is associated with increased energy intake and reduced satiety (33, 34). This may be due to fast eating interfering with satiety signals (35) or affecting the secretion of gastrointestinal hormones that regulate appetite (36), delaying the feeling of fullness.

One of the most intriguing findings of this study is the association between higher VFA and TrFM with both emotional overeating and emotional undereating, a relationship that has produced mixed results in previous research. While several studies, including ours, have reported a positive correlation between emotional overeating and childhood obesity (20, 37). However, other studies have not reported a significant relationship (38, 39). Part of the researches have even suggested gender differences in emotional behavior. In boys, body fat percentage and waist circumference are positively correlated with emotional overeating (40) but negatively correlated with emotional undereating (27, 40).

In contrast, our findings suggest that abdominal fat accumulation in children is positively correlated with emotional undereating. We speculate that several factors may contribute to these results. First, both emotional undereating and emotional overeating are manifestations of emotional eating behavior and may arise from shared environmental influences over the long term. Hence, a moderate positive correlation exists between them (41, 42). Children who tend to experience emotional undereating may also exhibit tendencies toward emotional overeating. Moreover, emotional undereating might stem from individuals suppressing their appetite in response to stress or anxiety rather than sustained undereating over time. This could disrupt their dietary patterns and rhythms (43). Once their mood improves, they may seek higher-energy foods to alleviate negative emotions, potentially leading to the accumulation of abdominal fat (44, 45). Additionally, children scoring high on emotional eating behavior scales may be more prone to prolonged exposure to negative emotions, resulting in increased cortisol levels and central obesity (46). Elevated cortisol levels can further stimulate appetite (47), contributing to the observed positive correlation between emotional undereating and emotional overeating. Indeed, a study noted that children with the highest weight tended to experience more emotional undereating (48).

Notably, our study also prospectively identified a significant relationship between children's eating behaviors and abdominal fat accumulation. In our results, we accounted for the random effect of school and controlled for factors such as age, sex, BMI, maternal education level, annual family income, CRF and intervention measures. We found that increases in VFA and TrFM in children were positively correlated with certain eating behaviors, namely, FR, EU and EO. Additionally, we observed that an increase in TrFM was associated with increased EF scores. These findings were consistent with those obtained from our cross-sectional analysis. Abdominal fat mass serves as an indicator of cardiovascular metabolic health in obese children (10). A recent Mendelian randomization study further underscored the association between higher childhood BMI and eating disorders such as emotional overeating (49). Emotional eating can lead to increased food intake and weight gain, perpetuating a cycle of dissatisfaction with weight and further overeating (50). Our results aligned with these findings, demonstrating a positive correlation between increases in VFA and TrFM in children and elevated scores in EU and EO. These findings suggested that emotional eating behaviors may contribute to the accumulation of visceral fat and overall adiposity in children. Overall, our study highlighted the importance of understanding the interplay between eating behaviors and abdominal fat accumulation in childhood, as it has implications for long-term metabolic health outcomes.

As previously documented, eating behaviors established during childhood tend to persist and influence adult eating habits (51). Inappropriate eating behaviors can contribute to obesity and related metabolic disorders. A prospective study involving 2,951 7-year-old children examined the relationship between eating behaviors and cardiovascular metabolic health at 10 years of age. Regression analysis revealed that eating behaviors at age 7 were predictive of cardiovascular metabolic risk at age 10. Notably, behaviors categorized as “food avoidance” (e.g., SE, SR) exhibited a protective effect, whereas “food approach” behaviors (e.g., FR, EF, EO) were associated with increased cardiovascular metabolic risk (52). Several prospective studies focusing on infants have consistently demonstrated that a robust appetite in early infancy plays a pivotal role in children's subsequent weight gain. For example, heightened food responsiveness in infancy has been linked to subsequent increases in BMI (53). Additionally, findings from one birth cohort revealed that increased eating delay and satiety at 3 months were significantly associated with lower BMI at 6 months (54). These results were corroborated by another prospective study (55). However, contrary to expectations, our study did not reveal a prospective relationship between increased abdominal fat accumulation in children and SE, SR, DD, or FU. These results may reflect the variances among children in Ningbo compared with those in other regions or that our study lacked the sensitivity to detect subtle changes over a short timeframe.

The strong associations between food responsiveness, emotional eating, and abdominal fat accumulation suggest that behavioral interventions targeting these specific eating behaviors may have a substantial impact on reducing visceral and trunk fat. While many obesity interventions focus on overall calorie intake and physical activity (56), our findings indicate the need for more tailored approaches that address emotional and behavioral aspects of eating. Programs that help children recognize and manage emotional triggers for overeating, for instance, may be particularly effective in reducing fat accumulation in high-risk fat areas.

This study has several limitations. First, the CEBQ assessments relied on parental reports, which may introduce bias on the basis of parents’ subjective perceptions of their children's eating behaviors. Additionally, the retrospective nature of the questionnaire data collection may introduce recall bias. Although adjustments were made for the influence of intervention factors in the analysis of follow-up data, some factors related to the intervention might have impacted the outcomes. However, in the OptiChild study, the intervention did not significantly affect CEBQ scores or body composition, suggesting that the influence of the intervention on the results of this study may be relatively minor. Moreover, this research focused on children aged 8–10 years, limiting the generalizability of the findings to other age groups and populations. Future research should consider expanding the age range to enhance the applicability of the results.

## 5 Conclusion

In conclusion, our findings provide novel insights into the complex relationships between eating behaviors and abdominal fat accumulation in children, highlighting the importance of addressing maladaptive eating patterns early in life to prevent obesity-related health risks. Our findings generally support the notion that childhood eating behaviors affect abdominal fat accumulation in children, in which “food proximity” behavior increases VFA and TrFM in children. However, the relationship between some eating behaviors and abdominal fat accumulation in children is still complex, which emphasizes the necessity of further research.

## Data Availability

The raw data supporting the conclusions of this article will be made available by the authors, without undue reservation.
